# Acute Inflammatory Mediators in Young Adult Patients with COVID-19 in Mexico

**DOI:** 10.3390/pathogens10081056

**Published:** 2021-08-20

**Authors:** Anahí Maldonado-Cabrera, Aracely Angulo-Molina, Ubydul Haque, Carlos Velazquez, Andrea S. Álvarez-Villaseñor, Karla J. Santacruz-Gómez, Ana L. Gallego-Hernández

**Affiliations:** 1 Departamento de Ciencias Químico-Biológicas, Universidad of Sonora, Hermosillo 83000, Mexico; a201203057@unison.mx (A.M.-C.); velaz2@unison.mx (C.V.); 2 Department of Biostatistics and Epidemiology, University of North Texas Health Science Center, Fort Worth, TX 76107, USA; mdubydul.haque@unthsc.edu; 3 Coordinación Médica de Investigación en Salud, Instituto Mexicano del Seguro Social, La Paz 23920, Mexico; andrea.alvarez@imss.gob.mx; 4 Departamento en Física, Universidad de Sonora, Hermosillo 83000, Mexico; karla.santacruz@unison.mx

**Keywords:** COVID-19, young adults, hematological profiles, coagulopathy

## Abstract

Young adults (18–40 years old) are an active population with high risk of infection and transmission of COVID-19. They are considered a low-risk population due to its low 1.0% case fatality rate (CFR). Despite their high clinical usefulness to prevent fatal cases, inflammatory and coagulation biomarkers studies are limited. For this reason, we performed a retrospective cohort study with COVID-19 patients in Hermosillo, Mexico, to assess inflammation, coagulopathy profile, and severity outcomes in young adults. We analyzed blood samples to determine the neutrophil/lymphocyte ratio (NLR), neutrophil/monocyte ratio (NMR), lymphocyte/monocyte ratio (LMR), platelet/lymphocyte ratio (PLR), and C-reactive protein (C-RP). We included epidemiological features and comorbidities, and compared them to the severity status. Only 359 COVID-19-confirmed young adults were included in the ambulatory (44.8%), hospitalized (42.9%), and death (12%) severity groups. Laboratory results showed an increase in NMR, LMR, and C-RP associated with the aggravated patients. Additionally, obesity, arterial hypertension, and type-2 diabetes mellitus (T2DM) were associated with the COVID-19 severity outcome. We found that 9.1% and 30.3% of young adults presented the novel COVID-19-associated coagulopathy (CAC) and the risk of CAC, respectively. These parameters can be considered independent biomarkers reflecting an enhanced inflammatory process related to the COVID-19 prognosis.

## 1. Introduction

Severe acute respiratory syndrome coronavirus 2 (SARS-CoV-2), has quickly spread around the world, causing the coronavirus disease 2019 (COVID-19) outbreak that has killed millions of people [1]. The common clinical features of COVID-19 include fever, dry cough, anorexia, malaise, dyspnea, fatigue, myalgia, headache, anosmia, ageusia, and diarrhea [2,3].

The severity of COVID-19 has been associated with various hematological alterations from in complete blood count (CBC) cell lines, such as lymphocytopenia, neutrophilia, thrombocytopenia, leukocytosis, and NLR, NMR, and LMR calculated ratios [2]. Other acute inflammatory mediators that change during SARS-CoV-2 infection are elevated levels of D-dimer, total bilirubin, lactic dehydrogenase (LDH), myoglobin, procalcitonin, interleukin-6, erythrocyte sedimentation rate, serum ferritin, and C-reactive protein (C-RP), among others [4]. Although vaccines have proven effective for COVID-19 prevention, there is no recognized antiviral treatment. For this reason, early diagnosis is one of the main priorities to protect vulnerable subgroups of the population against SARS-CoV-2 infections.

Severe cases can develop life-threatening complications such as acute respiratory distress syndrome (ARDS), disseminated intravascular coagulation (DIC), and the novel COVID-19-associated coagulopathy (CAC) [5,6]. Moreover, DIC is related to thrombotic complications and it is a major factor in increased mortality [7]. The main features of CAC include elevated D-dimer and fibrinogen, a decrease in platelet counts, and prolongation of prothrombin time [5,6]. Likewise, SARS-CoV-2 activates pro-inflammatory molecules that induce a multiorgan lesion, which includes direct viral toxicity, endothelial damage, thrombosis formation, and immune response dysregulation [8].

Elderly patients present a higher risk of complications, especially those with previous comorbidities such as type-2 diabetes mellitus (T2DM), cardio- and cerebrovascular diseases, and obesity, among others [2,9]. Nevertheless, young adults in Mexico also present a high prevalence of T2DM (1.4%), arterial hypertension (AH) (6.4%), and obesity (36.1%), which increase the vulnerability of this subgroup [10]. Case fatality rate (CFR) increases with age; up to 45% for patients over 80 years old, in contrast to under 1% for young adults (18–40 years old) [11]. Although young adults present a lower CFR, infection transmission has been mostly related to this group. Studies that evaluate the impact on young adults are scarce, despite being the most frequently infected population range and the last to be considered for preventive vaccination programs [11].

However, the limitations relate to the lack of data and little clinical information available for young adult patients with COVID-19. The most abundant information is focused on elderly patients, who represent the main population at risk, with a higher CFR [12]. This study aimed to identify changes in acute inflammatory and coagulopathy biomarkers during COVID-19 infection in patients from 18–40 years old. Our results have implications for biomarker selection for the different COVID-19 severity outcomes in young adults.

## 2. Results

### 2.1. Epidemiological Results

**Figure 1:**
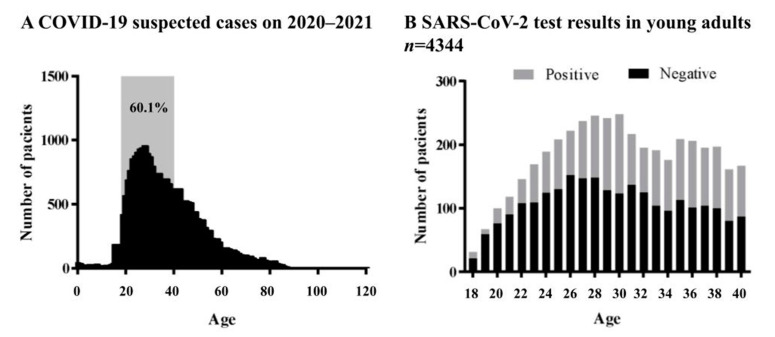
Suspected COVID-19 cases in Hermosillo, Sonora from March 2020 to March 2021. (**A**) Age distribution of all suspected COVID-19 cases. (**B**) RT-PCR or antigen test results of young adult patients.

A total of 30016 patients with suspected COVID-19 used the clinical IMSS services from March 2020 to March 2021; 60.1% of those cases corresponded to young adults (*n* = 18,040), described as patients from 18–40 years old (Figure 1A).

Of all suspected young adult patients, only 4344 were tested for SARS-CoV-2 (Figure 1B). Out of the total number of confirmed COVID-19-positive patients (*n* = 1681), the mean age was 31.1 years old and 44.3% were male. The overall case fatality rate (CFR) was 3.5%. For these patients, the most common clinical manifestations were headaches (81.7%), cough (71.1%), fever (68%), and myalgia (67%). The primarily underlying chronic disease found was obesity with a prevalence of (12.7%), followed by AH (8.6%) and T2DM (4.9%) (Table 1). In our study, there was a higher prevalence of young adults with T2DM compared to the Mexican and US international rates (1.4% and 4.3%, respectively) [13,14]. The demographic and epidemiological parameters were compared between the ambulatory, hospitalized, and death groups, showing a significant statistical difference in the T2DM, AH, and obesity patients (Table 1). These data suggest that these selected comorbidities in young adults can significantly affect the severity of the COVID-19 outcome.

### 2.2. Acute Inflammatory Mediators

**Figure 2:**
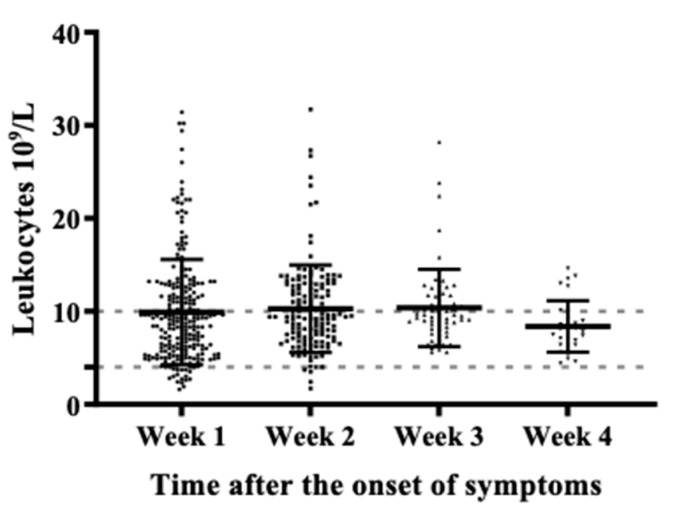
Dynamic analysis of leukocytes during the first four weeks after the onset of symptoms (*n* = 267). Leukocyte reference value: 4.6–10.2 × 10^9^/L [17]. The dotted line indicates a reference value.

For further and deeper analysis, we collected the medical and laboratory records of 359 patients during their COVID-19 convalescence; all of them were included for the inflammatory and coagulation profile analysis. Laboratory abnormalities in the complete blood count (CBC), particularly leukocytes count changes, allowed for checking the status of SARS-CoV-2 infection since the hematopoietic system suffers significant impacts during COVID-19 evolution [4,15]. The leukocyte count was analyzed during the first four weeks after the onset of the symptoms (Figure 2). Results showed an increase in leukocytes during week 1 (9.9 × 10^9^ /L), and week 2 (10.2 × 10^9^ /L), in response to the inflammation and infection of COVID-19 [16]. Leukocyte values returned to normal ranges (8.3 × 10^9^/L) during the fourth week, suggesting an already controlled inflammatory and disease process.

**Figure 3:**
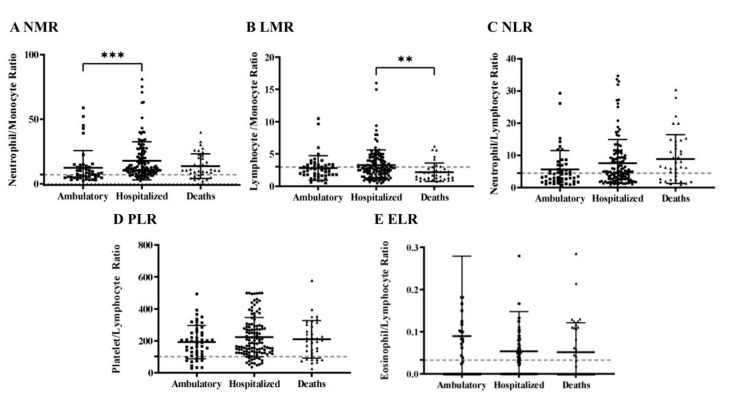
Ratio of biomarkers of leukocyte cellular lines of the ambulatory, hospitalized, and death patient groups. (**A**) Neutrophil/monocyte ratio (NMR) (*n* = 169; reference value: 7.2) [18]. (**B**) Lymphocyte/monocyte ratio (LMR) (*n* = 171; reference value: 3) [19,20]. (**C**) Neutrophil/lymphocyte ratio (NLR) (*n* = 173; reference value: 4.5) [19,20]. (**D**) Platelet/lymphocyte ratio (PLR) (*n* = 172; reference value: 120) [19,20]. (**E**) Eosinophil/lymphocyte ratio (ELR) (*n* = 174; reference value: 0.2) [21]. The dotted line indicates the reference value for each parameter. Statistical analysis was performed using one-way ANOVA and Kruskal–Wallis test: ** *p* < 0.01; *** *p* < 0.001.

The changes in the leukocyte count were further analyzed during the first two weeks after the onset of symptoms with five hematological ratios: neutrophil/lymphocyte ratio (NLR), neutrophil/monocyte ratio (NMR), lymphocyte/monocyte ratio (LMR), platelet/lymphocyte ratio (PLR), and the eosinophil/lymphocyte ratio (ELR). These ratios described the correlation between the hematological cell lines and indicated changes in the leukocyte cellular components. Furthermore, these ratios are biomarkers associated with poorer survival in acute lung bacterial infection, sepsis, and many solid tumors, including gastroesophageal, colorectal, pancreatic, prostate, and breast carcinoma, and COVID-19 [18,19]. Interestingly, in this study, NMR showed increased values, and statistical difference ratios between the ambulatory (12.5) and hospitalized (17.8) groups (Figure 3A). For LMR, a statistical difference between hospitalized (3.2) and death means (2.1) was found, clarifying the lower lymphocyte count in the death group (Figure 3B). Conversely, NLR, PLR, and ELR means (7.6, 218.3, and 0.06, respectively), showed no differences between the severity groups, however, they exhibited higher values than the ones expected for the reference values (dotted line) (Figure 3C–E).

**Figure 4:**
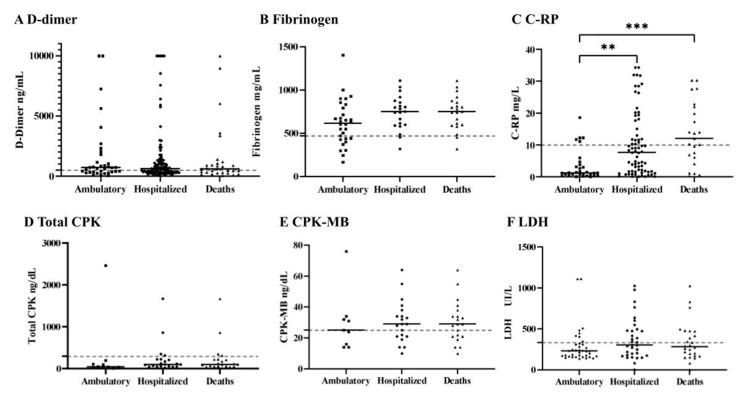
Acute inflammatory and coagulation biomarker analysis of the ambulatory hospitalized and death groups. (**A**) D-dimer (*n* = 112; reference value: 500 ng/dL) [17]. (**B**) Fibrinogen (*n* = 83; reference value: 471 mg/dL) [17]. (**C**) C-reactive protein C-RP (*n* = 119; reference value: 10 mg/L) [17]. (**D**) CPK (*n* = 64; reference value: 294 ng/dL) [17]. (**E**) CPK-MB (*n* = 55; reference value: 25 ng/dL) [17]. (**F**) LDH (*n* = 133; reference value: 333 UI/L) [17]. Dotted line indicates reference value for each parameter. One-way ANOVA and Kruskal–Wallis test: ** *p* < 0.01; *** *p* < 0.001.

The comparison between the COVID-19 severity groups, examining inflammatory biomarkers such as D-dimer, fibrinogen, LDH, C-RP, and cardiac markers, is shown in Figure 4. D-dimer is a specific degradation product that is produced by hydrolysis of fibrin, and it reflects the effect of COVID-19 on coagulopathy alterations [22]. In our study, the mean value for D-dimer was 1697 ng/mL for ambulatory patients, 1611 ng/mL for hospitalized patients, and 1533 ng/mL for the death group; values three times higher than the ones expected for reference values (dotted line). However, there were no statistical differences between the severity groups for this biomarker (Figure 4A).

Fibrinogen is a soluble protein and a fibrin precursor in blood plasma. In COVID-19 patients, fibrinogen concentrations are at the upper limits of normal, presumably as an acute phase response [16]. As expected, fibrinogen was found to be increased in all severity groups of young adult patients, but there were no significant differences between the COVID-19 severity groups (Figure 4B).

C-RP is an acute inflammatory phase protein produced by the liver and it increases in response to inflammation [20]. There were significant differences between the ambulatory (3.4 mg/L) compared with the hospitalized group (10.0 mg/L), and ambulatory versus death (13.4 mg/L) group (Figure 4C). This study showed significantly higher levels of C-RP in terms of the severity outcomes, which suggested that C-RP is a serum biomarker for disease aggravation in young adult COVID-19 patients.

CPK and CPK-MB are enzymes located in the muscle tissue, and their levels rise due to skeletal muscle damage and necrosis in rhabdomyolysis [23]. During COVID-19, the effect of these enzymes is unclear, however, the increased levels might be caused by viral myositis, immune hyperactivation, toxic effects of cytokines, or other mechanisms [24]. In COVID-19 young adult patients, CPK values were increased in the hospitalized (903 ng/dL), and the ambulatory (306 ng/dL) group (Figure 4D). Meanwhile, CPK-MB means were found to be increased in all the severity groups (Figure 4E). Interestingly, none of these damaged muscle biomarkers produced a statistical difference between the COVID-19 severity groups evaluated, however, it is important to mention that these two parameters had sample size smaller than expected for this study (*n* = 65).

LDH is a biomarker of glucose metabolism. It is produced in tissues throughout the body, catalyzes pyruvate to lactate, and is released from cells upon damage to their cytoplasmic membrane [25]. From these results, only hospitalized patients showed increased values. In other studies, LDH has been reported to be increased in COVID-19, pneumocystis, and influenza A (H1N1), which indicates that LDH elevation is relevant during lung injury [25].

### 2.3. Coagulopathy Alterations

COVID-19 is known to be frequently associated with coagulopathy and thrombotic events [6,26]. In our study, only 1.1% of the young adult patients with COVID-19 developed DIC according to the International Society for Thrombosis and Haemostasis (ISTH) criteria (Table 2). The prevalence in a general population report ranges from 4.0 to 6.2%, however, the lower prevalence could be due to the age differences [6]. Our results show that 9.1% of the total patients presented laboratory criteria for the novel CAC. Moreover, the risk of developing CAC increased up to 30.3% in all the patients. Remarkably, the three coagulopathies analyzed in this study (DIC, CAC, and risk of CAC), were associated with differences in the COVID-19 severity groups.

## 3. Discussion

Comorbidities have been strongly related to the seriousness of COVID-19. In our study, we found obesity as the most common comorbidity with a prevalence of 12.7% and a CFR of 3.5%; additionally, obesity, AH, and T2DM demonstrated an association with severe outcomes of COVID-19, and these values have similarities with previous reports of young adults (Table 1) [27,28,29]. Our results showed that T2DM presented a higher prevalence in COVID-19-confirmed young adults when comparing Mexican and the US populations [13,14].

The COVID-19 inflammation process during the initial stages of the disease might show a normal or decreased leukocyte count at the expense of the lymphocyte count. During the evolution of viral pneumonia, the balance of T cells, CD4 T cells, and CD8 T cells is crucial in the fight against pathogens and the development of autoimmunity. Furthermore, lymphocytes, neutrophils, and monocytes act dynamically to produce immune-mediated interstitial pneumonitis, with inflammation and activation of subsequent cytokines, such as IL-1, IL-6, IL-8, IL-21, TNF-β, and MCP-1, at the site of infection [16]. Therefore, ratios can change with the severity of COVID-19 [30]. In this study, NMR and LMR showed significant differences between the severity groups (Figure 3A,B). The NMR value corresponds to the progressive increase in neutrophils, and/or the decrease in monocytes. Elevated neutrophils often indicate that patients have a bacterial infection, and the infection is aggravated; in contrast, the decrease in monocytes means migration at the site of infection [8,16]. Similarly, high LMR means an increase in lymphocytes over monocytes. The NMR and NLR ratios were previously described as elevated among COVID-19-positive patients of the Mexican population, indicating a better efficiency to predict poor prognosis [18]. NMR values also differed significantly in the COVID-19 severity groups (Figure 3A,B). These data also show the importance of ethnic differences affecting inflammatory parameters. NLR also helps to predict prognosis in various pathological conditions [18,19]. NLR means the progressive increase in neutrophils and the decrease in lymphocytes [19]. Increases in NLR in patients with COVID-19 are highly associated with a poor prognosis [19,20,30].

D-dimer might reflect the effects of infection on coagulation in infectious diseases. High concentrations of D-dimer have been reported in patients with COVID-19, and it is a sign of the blood prothrombotic state and the presence of thrombosis; one of the main criteria to detect DIC and CAC alterations [5,6,22]. Additionally, high D-dimer is likely to be associated with coagulation disorders, microthrombotic formation, pulmonary embolism, and acute myocardial infarction in hospitalized and dead patients, and this medical complication can lead to hypoxemia, respiratory failure (ARSD), DIC, or death [22]. In this retrospective study, D-dimer values tripled beyond the normal parameters, but they did not significantly increase between the severity groups (Figure 4A). Our results differ from other published studies, which report that D-dimer is often associated with severe prognosis [22,27,31,32,33].

C-RP’s main mechanism of action is activating the complement and enhancing phagocytosis. In different studies, C-RP is correlated with the level of inflammation and it is found to be increased and associated with COVID-19 severity [20,34]. In our study, high C-RP levels differ statistically between the ambulatory, hospitalized, and dead patients (Figure 4C), which suggested that CRP is a marker of disease aggravation in COVID-19 patients; these results stand accordingly with other published studies [34].

An increase in total CPK and CPK-MB cardiac and muscle biomarkers has been reported based on the infection-induced metabolic demand and the viral lesions of the myocardial tissue, among other causes [23]. In our results, only CPK-MB was elevated in comparison to other publications (Figure 4D,E) [23,35,36]. Clinically, the prognostic value is especially relevant in the follow-up of patients with risk factors, such as AH, T2DM, and previously documented cardiovascular disease [23].

Severe forms of COVID-19 display coagulation abnormalities that have been associated with respiratory deterioration and death; the most important form of coagulopathy is DIC [6]. Unfortunately, DIC contributes to a sudden deterioration of pulmonary oxygen exchange in patients with COVID-19 infections [5,36]. In contrast to other studies, we found that only 1.1% of young adults presented DIC, which can be explained by the difference in the population age [36]. In addition, COVID-19 has specific clinical and laboratory features that are distinctly different from the ‘classical’ presentation of DIC [6]. For this reason, COVID-19-associated coagulopathy (CAC) is a new criterion for describing major alterations presented in COVID-19 patients. In our study, we found that 9.1% of all the patients showed CAC. Compared to previous publications, CAC was found in 10% of patients during hospital admission [5,6].

The risk of developing CAC was described by three criteria. Unfortunately, we could not examine two of them due to the retrospective design of the protocol and because they were not performed routinely. However, the third criterion was the fibrinogen biomarker, one of the habitual laboratory analyses at the IMSS [5]. Therefore, the risk of CAC was integrated considering the earlier criterion of CAC and the increased fibrinogen values. Despite this limitation, the risk of CAC was found in 30% of all the COVID-19 patients. Interestingly, all the coagulopathy alterations presented associations with the COVID-19 severity status. This is important since the identification of coagulopathy abnormalities can help assess the final clinical prognosis, which is needed for further investigation. The major strengths of this study include a large number of observations (*n* = 359), and high representativeness of the study population since different clinical centers of primary care and hospitals were involved.

## 4. Materials and Methods

### 4.1. Data Sources

A retrospective cohort study was designed to analyze the severity of COVID-19, by calculating the prevalence of death (5%) and hospitalization (14%) as severity statuses among COVID-19 patients in a representative Mexican population [18]. With a confidence level of 95% and an accuracy of 10%, a total of 65 patients were required in each group. The data were collected at the Instituto Mexicano del Seguro Social (IMSS) in Hermosillo, Sonora, Mexico. From March 2020 to March 2021, we gathered patients from 18–40 years of age and who were confirmed to have COVID-19 with a Berlin quantitative reverse transcription polymerase chain reaction (qRT-PCR) or an Abbott COVID-19 antigen test. This study was approved by the local Ethics and Investigation Committee at IMSS (Registration Number R-2021-2604-024; Approval date: 14 April 2021) and carried out following the national and international ethical directives for conducting research in humans such as the Declaration of Helsinki, as well as those established in the Good Clinical Practice Guidelines. Confidentiality and privacy of all collected information were assured.

A proper medical history and detailed clinical examination were conducted for each patient. The laboratory tests included complete blood count (CBC), blood biochemistry, myocardial biomarker, C-reactive protein, and coagulation profile. These analyses were based on the clinical condition and the medical attention level at the IMSS. Only laboratory results obtained during the first two weeks after the onset of symptoms were selected for analysis unless otherwise indicated.

### 4.2. Exposure Variable

Epidemiological variables included age, gender, and history of comorbidities such as T2DM, AH, obesity, and asthma, among others. The hematological biomarkers included cellular counts and ratios to compare the proportion of primary hematological cell lines, neutrophil/lymphocyte ratio (NLR), neutrophil/monocyte ratio (NMR), lymphocyte/monocyte ratio (NMR), and platelet/lymphocyte ratio (PLR), as well as acute inflammatory and coagulation parameters such as C-reactive protein (C-RP) and myocardial biomarkers. 

### 4.3. Outcome Measures

Based on the severity of the disease, all patients were classified into three groups: ambulatory, hospitalized, and death. The ambulatory group included mild and moderate cases presented at the main primary care IMSS facilities. The hospitalized group consisted of severe to critical cases which fulfilled hospitalization criteria with two or more of CURB65, a chest X-ray image showing the progression of COVID-19 infiltrations, distress or respiratory failure, shock, or organ failure [37]. The death group was all hospitalized patients with signs of deterioration whose final prognosis was fatal.

Disseminated intravascular coagulation (DIC) was calculated according to the ISTH scoring system [38]. The novel COVID-19-associated coagulopathy (CAC) was determined based on two or more of the following criteria: (1) decrease in platelet count (less than 150 × 10^9^ /L); (2) increase in D-dimer (more than two times the upper limit of normal); (3) >1s prolonged prothrombin time or international normalized ratio (INR) > 1.2; (4) decrease in fibrinogen level; and (5) thrombosis [5]. The risk of CAC is defined as one of the CAC criteria, in addition to one of the following indicators: (1) increase in fibrinogen level, (2) increased von Willebrand factor (VWF), and (3) presence of lupus anticoagulant and/or high-titer antiphospholipid antibodies [5].

### 4.4. Statistical Analysis

The statistical software GraphPad Prism version 9.0.0 for Windows was used for data analysis. All data are presented as means and SD. Chi-squared and Fisher’s exact tests were used to compare the differences between the categorical groups. One-way ANOVA and Kruskal–Wallis tests were used to determine the differences of each laboratory parameter between the COVID-19 severity groups.

## 5. Conclusions

In this study, we found obesity as the most common comorbidity with a prevalence of 12.7% and a CFR of 3.5% in young adults with COVID-19 in Hermosillo, Sonora, Mexico; additionally, obesity, AH, and T2DM demonstrated associations with severe outcomes. Additionally, 359 young adult patients confirmed as having COVID-19 showed changes in hematological parameters (NMR, LMR, and C-RP), reflecting an enhanced inflammatory process related to an increase in the COVID-19 severity. Therefore, health care institutions should pay close initial attention to identifying progression biomarkers, to better guide treatment strategies and early assessment of the severity of COVID-19 in young adults.

## Figures and Tables

**Table 1:** Association analysis of chronic comorbidities with the different severities of outcomes of COVID-19 patients (ambulatory, hospitalized, and death), in Hermosillo, Sonora.

	Total Patients *n* (%)	Ambulatory *n* (%)	Hospitalized *n* (%)	Deaths *n* (%)	*p*-Value
Obesity	214 (12.7%)	136 (63.6%)	56 (26.2%)	22 (10.3%)	<0.000 ***
AH	144 (8.6%)	96 (66.7%)	35 (24.3%)	13 (9.0%)	<0.000 ***
T2DM	83 (4.9%)	41 (49.4%)	25 (30.1%)	17 (20.5%)	<0.000 ***
Smoking	82 (4.9%)	64 (78.0%)	15 (18.3%)	3 (3.7%)	0.304
Asthma	81 (4.8%)	70 (86.4%)	7 (8.6%)	4 (4.9%)	0.424
Pregnancy	44 (2.6%)	35 (79.5%)	9 (20.5%)	0 (0%)	0.153
Total	1681 (100%)	1407 (83.7%)	215 (12.7%)	59 (3.5%)	

Chi-square test: ⁎⁎⁎ *p* < 0.001.

**Table 2:** Association analysis of the coagulopathy alterations: disseminated intravascular coagulation (DIC), the novel COVID-19-associated coagulopathy (CAC), and the risk of CAC with the outcome severity of COVID-19 patients in Hermosillo, Sonora.

	Total *n* (%)	Ambulatory *n* (%)	Hospitalized *n* (%)	Deaths *n* (%)	*p*-Value
Disseminated intravascular coagulation (DIC)	4 (1.1%)	0 (0%)	3 (75%)	1 (25.0%)	0.0297 *
COVID-19-associated coagulopathy (CAC)	33 (9.1%)	4 (12.1%)	20 (60.6%)	9 (27.3%)	<0.0001 ***
Risk of CAC	109 (30.3%)	21 (19.3%)	69 (63.3%)	19 (17.4%)	<0.0001 ***
No alterations	213 (59.3%)	136 (63.8%)	62 (29.1%)	15 (7.0%)	
Total	359 (100%)	161 (44.8%)	154 (42.9%)	44 (12%)	

Chi-square test: * *p* < 0.05; *** *p* < 0.001.

## Data Availability

Not applicable.
